# A Three‐Phase Electrostatic Clutch with Variable Mechanical Impedance Control for Soft Robotic Systems

**DOI:** 10.1002/advs.202510291

**Published:** 2025-09-16

**Authors:** Dongyoung Lee, Heejin Yu, Joonbum Bae

**Affiliations:** ^1^ Electronics and Telecommunications Research Institute (ETRI) Daejeon 34129 South Korea; ^2^ Dept of Mechanical Engineering Ulsan National Institute of Science and Technology (UNIST) Ulsan 44919 South Korea; ^3^ School of Mechanical Engineering Korea University Seoul 02841 South Korea

**Keywords:** three‐phase electrostatic clutch, damping effect control, mechanical impedance control, phase modulation method

## Abstract

Various studies have explored mechanical impedance control strategies in soft actuators to enhance stability and precision, as the nonlinear characteristics of soft materials frequently lead to unwanted oscillations. Among these approaches, electrostatic (ES) clutches have shown promise for achieving friction‐based mechanical impedance control; however, conventional ES clutches are limited by issues such as residual charge accumulation and stick‐slip behavior, which compromise force stability and reliability over time. To address these limitations, this study introduces a three‐phase ES clutch with variable mechanical impedance control, designed to enable continuous friction modulation and thereby reduce force degradation and oscillatory motion. Experimental validation was conducted through tensile tests and mechanical impedance applications in a vibration system. These findings suggest that the proposed three‐phase ES clutch offers a robust and adaptable solution for friction modulation and mechanical impedance in soft robotic and wearable systems, effectively addressing the key limitations of conventional ES clutch technology.

## Graphical abstract

The research introduces a three‐phase electrostatic (ES) clutch with variable mechanical impedance control, specifically designed to enable continuous and precise friction modulation, thereby reducing both force degradation and oscillatory motion. This method effectively resolves conventional ES clutch issues, such as residual charge accumulation and stick‐slip behavior, which significantly compromise force stability and operational reliability.

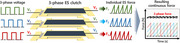

## 1 Introduction

Soft robotic systems utilizing soft actuators face significant challenges due to the nonlinear properties of soft materials, leading to oscillatory behavior and imprecise control in various applications.^[^ ^1^, ^2^, ^3^, ^4^, ^5^, ^6^, ^7^ ^]^ These challenges are particularly problematic in dynamic tasks, where the actuator's response becomes unpredictable and inconsistent. Although advanced control algorithms have been developed to mitigate these issues, they often do not address the inherent non‐linear dynamics of soft materials. Integrating mechanical impedance structures could significantly enhance stiffness regulation and stability.^[^ ^8^, ^9^, ^10^, ^11^, ^12^ ^]^ However, despite technological advances, achieving reliable mechanical impedance control in soft robotics remains a critical challenge.

Traditional approaches to mechanical impedance control have utilized piston dampers and electromagnetic dampers, which are effective in vibration suppression and damping enhancement.^[^ ^13^, ^14^, ^15^, ^16^ ^]^ However, their rigid and heavy structures significantly hinder compatibility with soft actuators, compromising flexibility and compliance. To overcome these limitations, elastic fluidic variable impedance systems have been developed, utilizing fluids whose viscosity changes in response to magnetic or electric fields. Among these, magnetorheological (MR) fluid actuators are commonly used to achieve variable stiffness, especially in applications like wrist rehabilitation.^[^ ^17^, ^18^, ^19^, ^20^ ^]^ However, their heavy and bulky designs make them impractical for lightweight and adaptive soft robots. Similarly, shape memory polymers (SMPs) and low melting point alloys (LMPAs) can induce stiffness changes through heat, but slow cooling times hinder real‐time control.^[^ ^21^, ^22^, ^23^ ^]^ Additionally, pneumatic actuators have been employed for variable impedance control using air pressure and friction mechanisms. Techniques such as pneumatic layer jamming and particle jamming enable dynamic stiffness adjustment.^[^ ^24^, ^25^, ^26^ ^]^ However, these methods often suffer from vibration issues during operation and delays caused by pumps and valves, making precise and rapid control difficult. Recent advances in smart‐material‐based soft actuators have demonstrated various approaches to vibration attenuation. For example, stiffness‐tunable electroadhesive pads, dielectric elastomer actuators (DEAs) for dynamic modulation, and triboelectric‐based dampers have been introduced.^[^ ^27^, ^28^, ^29^ ^]^ However, these approaches do not enable direct mechanical impedance control, as they rely on passive friction enhancement or stiffness modulation, and lack of active friction control.

To overcome the limitations of previous approaches, electrostatic (ES) clutches have emerged as a promising technology for soft actuators. These clutches generate electrostatic forces through the accumulation of opposite charges between two films, allowing friction control through voltage modulation.^[^ ^30^, ^31^, ^32^, ^33^, ^34^, ^35^, ^36^ ^]^ Due to their fast response, simple structure, energy efficiency, and high force generation.

**Figure 1:**
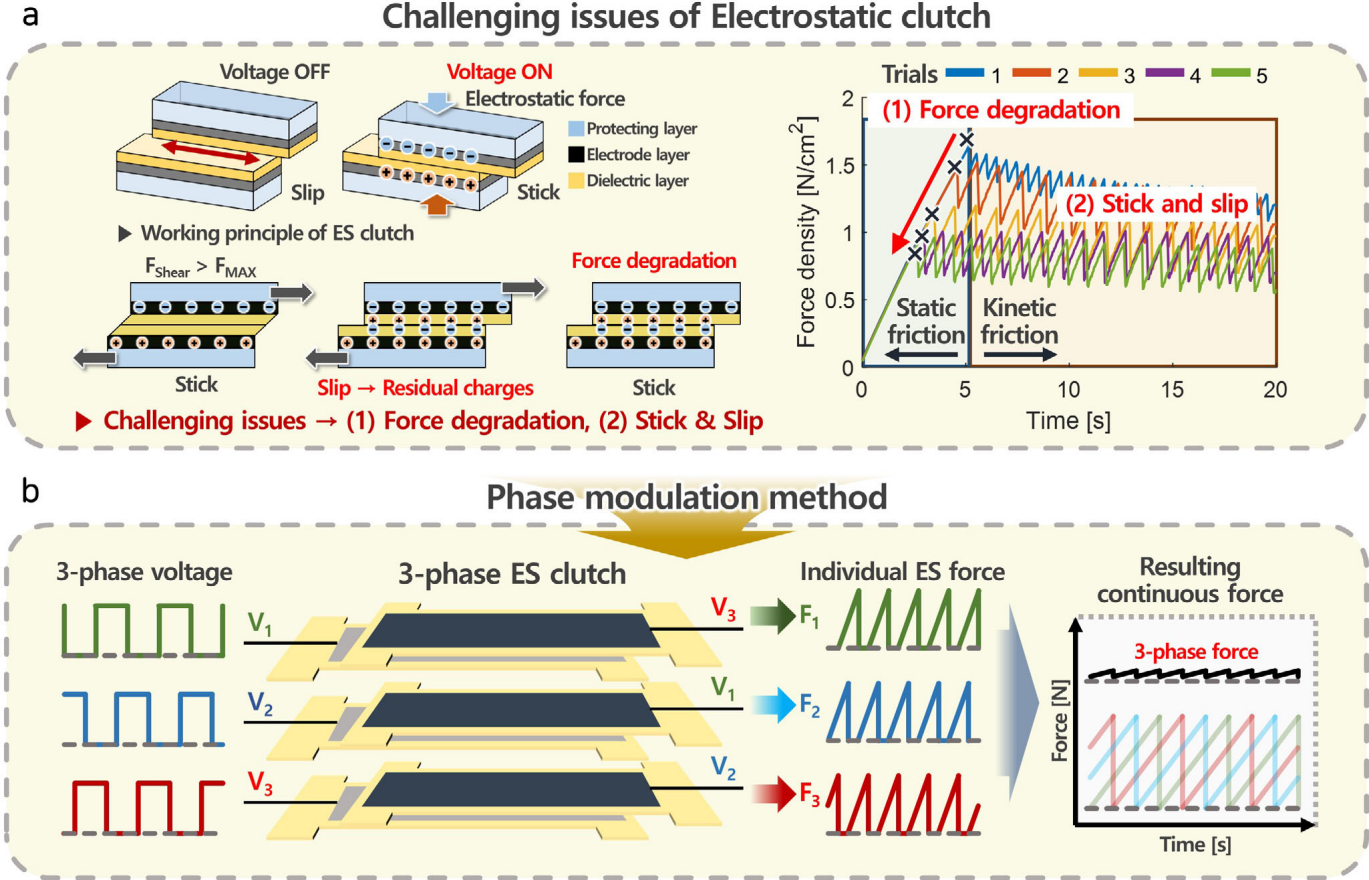
Overall process of three‐phase voltage and three‐phase force generation. a) Working principle and degradation of force and stick‐slip issues of the ES clutch. b) The proposed concept of the three‐phase ES clutch with the phase modulation method.

Despite these promising characteristics, the implementation of mechanical impedance control using ES clutches remains highly challenging. A primary difficulty lies in accurately characterizing force behavior in the kinetic friction state. Several studies have demonstrated friction or stiffness modulation using ES clutches.^[^ ^37^, ^38^, ^39^ ^]^ However, these studies did not address the long‐term repeatability of mechanical impedance control in the kinetic friction state. To address this, **Figure** **1**a shows the conventional ES clutch features during the pull in test. The primary challenges associated with ES clutches can be summarized as follows:

1) Force degradation from residual charge: Continuous application of DC voltage often leads to residual charge accumulation, gradually degrading performance during repeated use. To address this, various studies have proposed using AC voltage to reverse polarity and reduce residual charge accumulation.^[^ ^40^, ^41^, ^42^, ^43^, ^44^ ^]^ Additionally, some research has employed deionization treatments on the dielectric layer surface to remove residual charges after DC voltage experiments.^[^ ^45^ ^]^
2) Stick‐slip behavior: Another critical challenge is stick‐slip behavior, which significantly compromises precise mechanical impedance control. This phenomenon often arises when using AC voltage, as the alternating polarity induces oscillatory friction forces, leading to unsteady clutch performance.^[^ ^35^, ^43^, ^46^ ^]^ Even with high‐frequency AC, stick‐slip issues persist, causing instability, and reduced control precision.

To further reduce stick‐slip effects, some studies have proposed rigid structural modifications.^[^ ^47^, ^48^ ^]^ However, these rigid designs conflict with the soft and flexible nature of soft robotics, ultimately reducing compliance and adaptability. To address these challenges, a new control methodology specifically designed for mechanical impedance control using ES clutches is essential.

This study introduces a novel three‐phase ES clutch employing a phase modulation method (Figure 1b) The proposed clutch draws inspiration from three‐phase brushless DC (BLDC) motor concepts, achieving continuous stiffness modulation by sequentially energizing individual electrodes. This configuration significantly reduces nonlinear characteristics and maintains stable force generation, overcoming the inherent limitations of conventional single‐phase or AC voltage methods. The key innovation of this study lies in the systematic application of three‐phase control to ES clutches, which has not been previously attempted. By maintaining continuous voltage modulation, the system achieves dynamic stiffness control with significantly reduced nonlinearity, thereby overcoming the long‐standing challenges that have hindered practical implementation in soft robotics.

To validate the proposed concept, comprehensive experiments were conducted, including comparative analysis with conventional DC and AC voltage methods. The results demonstrate that the three‐phase ES clutch significantly outperforms existing approaches, particularly in terms of active stiffness control and damping coefficient adjustment, which are critical for enhancing the dynamic performance of soft actuators. Additionally, the potential of the proposed system as a force modulation component in wearable robotic applications was demonstrated, highlighting its versatility and practicality.

## 2 Results

### 2.1 Force Degradation and Stick‐Slip Issue

#### 2.1.1 Residual Charge and Friction Control

As shown in Figure 1a, an ES clutch consists of a dielectric layer, an electrode layer, and a protective layer. The dielectric layer prevents the flow of current, the electrode is used to apply voltage to form the static charge, and the protective layer enhances the durability. A set of ES clutches generates varying charges between layers, producing ES forces that induce friction. To enhance the functionality of the ES clutch, control should remain effective even in the slip state. However, limitations in control mechanisms have traditionally restricted their application to mainly function as linear brakes in static states, where friction control is managed using DC and AC voltages. The ES clutch shows the maximum static friction, which can be modulated by adjusting the applied voltage. However, once kinetic friction is triggered, force control becomes challenging due to limitations inherent to the ES clutch. In controlling friction during the slip state, two principal challenges are encountered: the first is the force degradation attributed to the residual charge. Repeated tensile tests on ES clutches have shown a decrease in the maximum ES force value in successive trials. Second, the nonlinear characteristics of the materials lead to a stick‐slip issue, where unsteady movement makes precise control in the slip state challenging.

The contact charging mechanism by which the residual charge is generated in the ES clutch is depicted in Figure S1 (Supporting Information). Throughout the activation of the ES clutch, friction‐induced charge transfer was observed; friction between the surfaces of the same dielectric layers results in charge movement due to contact electrification.^[^ ^49^, ^50^, ^51^, ^52^ ^]^ Residual charges that remain on the surface of the ES clutch generate residual ES forces. These forces persist even after completion of the ES clutch tensile tests and remain present even in the absence of voltage. The presence of these residual forces when the voltage is off is undesirable, as it hinders the free movement of the ES clutch. This poses significant challenges in the control and effective management of the ES clutch.^[^ ^42^, ^53^, ^54^, ^55^ ^]^

#### 2.1.2 Force Degradation and Stick‐Slip Issues

**Figure 2:**
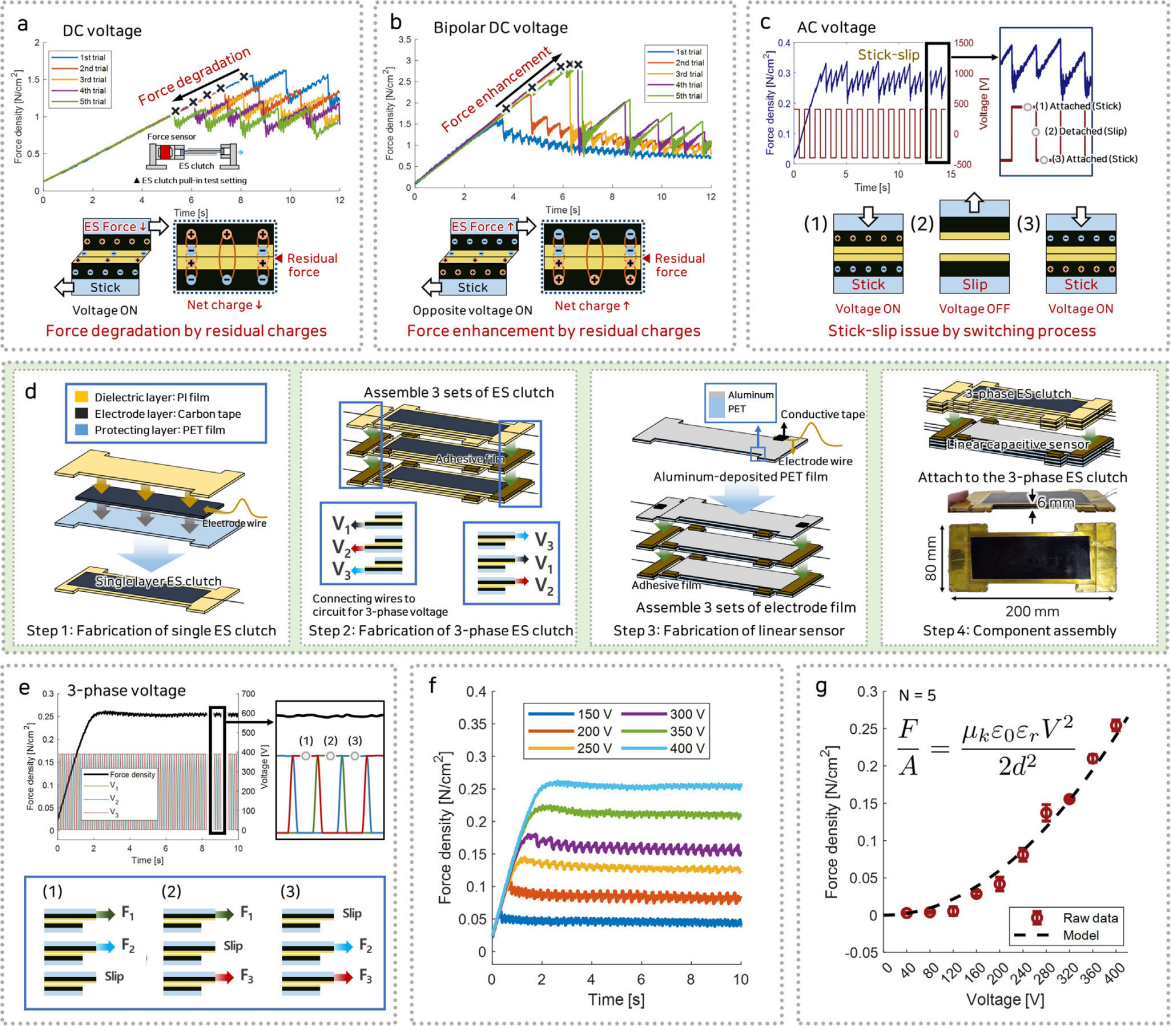
Pull‐in test results showing force degradation and stick‐slip issues under DC and AC voltage application, and improved performance of the three‐phase voltage application. a–c) Stick‐slip issue and force degradation with different voltage application. a) DC voltage application. b) Bipolar DC voltage application. c) AC voltage application. d) Fabrication process of three‐phase ES clutch with linear capacitive sensor. e–g) three‐phase ES clutch force performance. e) Improved force response under three‐phase voltage application. f) Constant force response over time for different voltages. g) Friction model and measurement results with respect to voltage.

To assess the characteristics of the ES clutch, a tensile test was performed using a customized pulling tester with a force sensor. **Figure** **2**a represents the stick‐slip issue and force degradation with the application of DC voltage to the ES clutch for repeated tensile test. The ES clutch was attached by the ES force, as the application of a DC voltage causes opposite charges to accumulate on each electrode layer. When a shear force was applied to an ES clutch, the surface detaches after reaching the maximum shear force. The ES force then causes the surface to reattach, creating a cycle of contact charging. This repetitive stick‐slip led to the generation of residual charges. The different polarities of the residual charge on the dielectric film and the applied charge on the electrode result in a net charge reduction. Therefore, repeated operations that involve contact charging and the accumulation of residual charges cause a force degradation issue.

The residual force exerted on the ES clutch varies according to the direction of the input voltage during each test. Following contact charging and the subsequent accumulation of residual charges, the application of a DC voltage in the opposite direction to that previously applied alters the system's behavior (Figure 2b). When the polarity of the applied voltage alternates during successive tests, both the applied voltage and the residual charge within the dielectric film align with identical polarities. As a result, additional charges of the same polarity as the residual charges accumulate, increasing the net charge on the electrode. This alignment intensifies the electric field across the dielectric film, thereby enhancing the ES force. Consequently, repetitive cycles of contact charging and residual charge accumulation contribute to a progressive increase in the ES force.

To address the issues that arise from the application of DC voltage in the ES clutch, previous research has explored the use of AC voltage. In Figure 2c, the results of the friction measurement under the influence of AC voltage are illustrated. With AC voltage, the voltage is set to an off state before detachment, causing the external electric field to disappear and subsequently eliminating contact charging. As a result, the effect of residual charge does not occur under AC voltage, thus maintaining maximum friction without force degradation for repeated tests. However, the occurrence of stick‐slip phenomena remains a challenge in friction control. Although AC voltage control systems are used to prevent residual charges, instances arise in which a reduction in force is observed, attributable to the transient absence of ES force during tests.

### 2.2 System Configuration

#### 2.2.1 Three‐Phase ES Clutch Configuration

The operation of the ES clutch with DC and AC voltage application shows force degradation and stick‐slip issues. To address these issues, a three‐phase ES clutch with three‐phase voltage was developed. The three‐phase ES clutch consists of three individual ES clutch units, each equipped with electrodes designed for independently applied voltages, enabling three‐phase voltage operation. As illustrated in Figure 2d, the fabrication process begins with the development of single ES clutch films, each comprising a protective layer, an electrode layer, and a dielectric layer, with electrode wires connected to facilitate charge transfer. The material selection criteria for the ES clutch were determined based on the functional requirements of each layer. For the dielectric layer, both the dielectric constant and thickness were considered. A polyimide (PI) film was selected due to its high wear resistance under repeated frictional contact and its low elasticity. In elastic materials, detachment does not occur simultaneously across the entire interface but progresses locally due to interfacial crack propagation.^[^ ^32^, ^56^ ^]^ To suppress such fracture‐driven failure and maintain stable shear contact during kinetic friction, the use of a compliant PI film helps to prevent abrupt interface separation.

The electrode layer, responsible for charge delivery to the dielectric surface, also required adequate adhesion. A carbon‐based conductive adhesive tape was employed to satisfy both electrical and mechanical compatibility. To enhance the structural stability of the thin and compliant dielectric and electrode layers, a moderate thickness polyethylene terephthalate (PET) film was used as a protective layer, providing mechanical support while retaining flexibility. These individual clutches are then assembled into a multilayer structure by stacking three units using a double‐sided adhesive film for secure bonding. Finally, a linear capacitive sensor layer is integrated to measure the linear displacement of the clutch, with three sets of sensor films fabricated similarly to the individual clutches and adhered to the assembly using the same adhesive technique. The interaction among the three sets of ES clutches across their individual layers allows each ES clutch to include a dielectric layer on only one side. This configuration facilitates independent regulation of the ES force for each clutch, eliminating interference from the other clutch films.

#### 2.2.2 Force Model

The kinetic friction (*F*_*shear*_) produced in a three‐phase ES clutch is calculated using a Coulomb friction model combined with a Maxwell pressure model‐based ES force equation:^[^ ^33^ ^]^

(1) $$F_{shear}=\mu_{k}F_{normal}=\mu_{k}\frac{\varepsilon_{0}\varepsilon_{r}V^{2}A}{2d^{2}}$$

In this model, the ES force is influenced by the dielectric constant of the layer (ϵ_*r*_), film thickness (*d*), kinetic coefficient of friction (µ_*k*_), applied voltage (*V*), and contact area (*A*). The dielectric constant reflects the medium's influence on the electric field, with higher values resulting in greater ES force. Similarly, the ES force is inversely proportional to the square of the film thickness. For this study, a PI film was selected due to its dielectric constant of 3.4 and a thin thickness of 5 µm. To evaluate friction during slippage, the µ_*k*_ of the PI film was utilized. The µ_*k*_, measured using the ASTM D‐1894‐90 method, was found to be 0.15. A maximum voltage of 400 V, below the dielectric breakdown voltage of the PI film, was selected to prevent damage to the ES clutch. The contact area significantly impacts the ES force. During operation, the effective contact area corresponds to the overlapping region of two active ES clutch sets. For this study, the actual contact area was 60 *cm* ^2^ per set, resulting in an active area of 90 *cm* ^2^ for performance measurements.

To assess the three‐phase ES clutch's performance, a tensile test was conducted to measure kinetic friction. Figure 2e presents the force response during the three‐phase voltage activation process. Unlike the AC voltage application method, two of the three layers are activated during the switching process, preventing force degradation and stick‐slip issues. Figure 2f presents the results of friction performance measurements for the three‐phase ES clutch under varying applied voltages. A power supply and high‐voltage converter were utilized to evaluate performance, with the applied voltage incrementally increased in 50 V steps from 50 to 400 V. The friction initially reached its maximum value and, despite minor stick‐slip effects over time, remained stable at a constant level. Figure 2g illustrates the maximum friction values extracted from each experimental dataset at varying voltages. The results demonstrated a quadratic relationship between the applied voltage and the friction, achieving a maximum of 22 N at 400 V. The measured friction values exhibited strong correlation with theoretical predictions derived from the Maxwell pressure model.

#### 2.2.3 Actuation Mechanism

**Figure 3:**
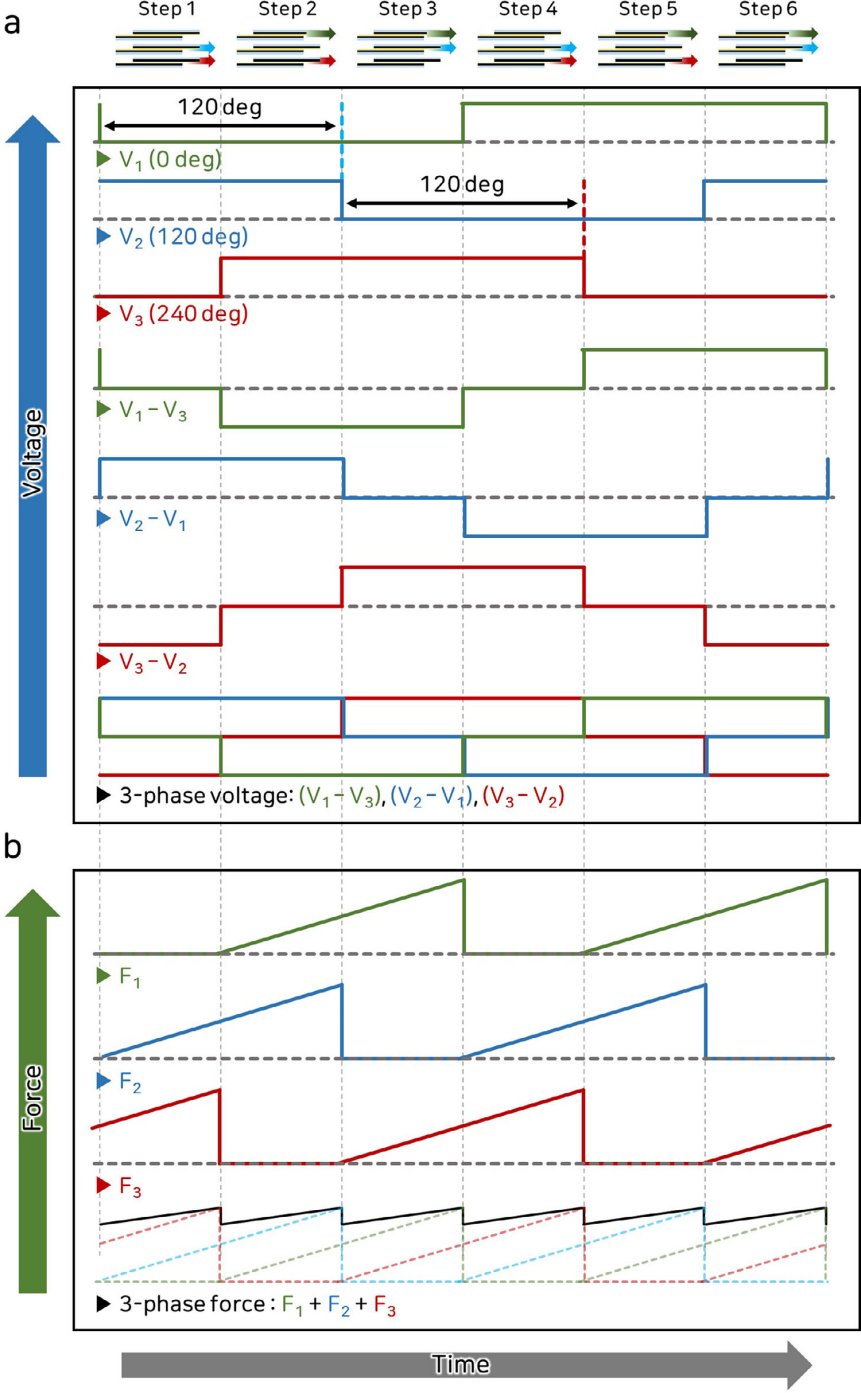
Activation process of the three‐phase ES clutch using phase modulation. a) Process of applying three‐phase voltage using phase modulation. b) Process of applying three‐phase force using three sets of ES clutches.

**Figure** **3**a and **Table** **1** illustrate the three‐phase voltage modulation process used to control the friction of the three‐phase ES clutch. Each of the three electrodes is driven by a monopolar square wave signal with a constant phase shift. This signal alternates between a constant applied voltage (+*V*) and zero voltage. The three‐phase waveform applied to the electrodes generates varying potential differences among them. The entire activation process consists of a six‐step cycle, with each step characterized by a distinct arrangement of potential differences. Additional explanations regarding the real‐time voltage measurements at both the circuit and the ES clutch are provided in Figure S2 (Supporting Information). During the cycle, the three‐phase ES clutch sequentially receives voltages *V*_1_, *V*_2_, and *V*_3_ with a phase difference of 120 degrees. The three sets of ES clutch repeat the voltage cycle ON / OFF sequentially, with six electrodes interconnected in a bipolar square wave configuration (*V*_1_ − *V*_2_, *V*_2_ − *V*_3_, *V*_3_ − *V*_1_). When the signals are applied simultaneously to the electrodes, a three‐phase voltage pattern emerges. The summation of these signals ensures that, at any given time, two of the ES clutches are in the ON state while the other is in the OFF state, thereby maintaining a consistent ES force.

**Table 1:** three‐phase voltage steps and their corresponding forces during the activation of the three‐phase ES clutch.

Step	*V*_1_	*V*_2_	*V*_3_	*V*_1_ − *V*_3_	*V*_2_ − *V*_1_	*V*_3_ − *V*_2_	Force
1	0	*V*	0	0	*V*	−*V*	*F*_2_ + *F*_3_
2	0	*V*	*V*	−*V*	*V*	0	*F*_1_ + *F*_2_
3	0	0	*V*	−*V*	0	*V*	*F*_1_ + *F*_3_
4	*V*	0	*V*	0	−*V*	*V*	*F*_2_ + *F*_3_
5	*V*	0	0	*V*	−*V*	0	*F*_1_ + *F*_2_
6	*V*	*V*	0	*V*	0	−*V*	*F*_1_ + *F*_3_

Figure 3b illustrates the frictional characteristics of a three‐phase ES clutch. In the off‐voltage state, each clutch undergoes slip, with phase differences among the clutches generating stick‐and‐slip effects throughout the system. When a three‐phase voltage is applied, the clutches sequentially transition between stick and slip states according to the voltage phase. This sequential activation compensates for the nonlinear motion caused by individual slip events, enabling the summation of forces to produce a stable and continuous output. Therefore, the amplitude of the applied voltage can manipulate the mechanical impedance. More detailed information on the overall system signal process is provided in Figure S3 (Supporting Information), and the circuit for the three‐phase ES clutch is described in detail in Figure S4 (Supporting Information). The stacking of multiple layers of the ES clutch allows the development of two‐ and four‐phase ES clutches, as shown in Figure S5 (Supporting Information). However, the two‐phase ES clutch experiences force interruptions due to stick‐and‐slip dynamics, and the four‐phase ES clutch, which activates only two layers at a time, does not offer significant advantages over the three‐phase system. Consequently, the three‐phase ES clutch was selected as an effective design for consistent force regulation in the proposed configuration.

### 2.3 Performance Verification

#### 2.3.1 Characteristics of three‐Phase ES Clutch

**Figure 4:**
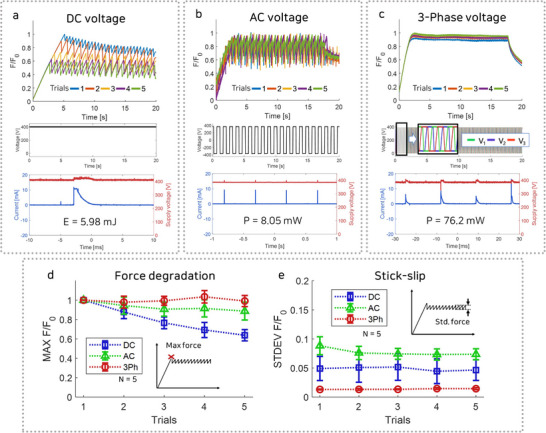
Performance comparison of the DC, AC, and three‐phase voltage applications. a–c) Measurement of the normalized force during five consecutive operations of the ES clutch, along with the applied voltage waveform, energy consumption, current, and supply voltage. a) DC voltage application. b) AC voltage application. c) Three‐phase voltage application. d) Force degradation performance measured as the maximum friction across repeated tests. e) Stick‐slip performance measured as the standard deviation of friction during slip across repeated tests.

The proposed three‐phase ES clutch was developed to address issues associated with conventional ES clutches activating method such as DC, AC voltage application. To ensure consistency, the same fabricated ES clutch was utilized for all experimental conditions involving DC, AC, and three‐phase voltage applications. To evaluate the influence of residual charge accumulation within the clutch, five repeated tensile tests were performed under each condition. The corresponding results are illustrated in **Figure** **4**a–c, and are summarized in Table S1 (Supporting Information). In Figure 4a, the force values are normalized by dividing the measured force by the maximum force of the initial experiment, designated as the reference force (*F*_0_), enabling direct comparison of the force characteristics. To analyze the effects of applied voltages on force degradation and stick‐slip phenomena, a graph was utilized to compare the characteristics.

First, to examine the issue of force degradation, the maximum normalized friction (MAX *F*/*F*_0_) was measured, as shown in Figure 4d. When DC voltage was applied, repeated experiments showed force degradation, with the normalized maximum force dropping to 0.65. This reduction is attributed to contact charging during the test, leading to the accumulation of residual charges that negatively influenced subsequent experiments. In contrast, upon application of AC and three‐phase voltages, the electric field was removed before contact charging occurred. This effectively prevented the accumulation of residual charges and maintained the normalized maximum force at 0.85 and 0.99 for AC and three‐phase voltage, respectively. As a result, the maximum friction remained stable during repeated tests.

Second, the stick‐slip phenomenon was investigated by measuring notable vibrations using the standard deviation, as shown in Figure 4e. To quantitatively assess stick‐slip effects, the normalized standard deviation of the force values (STDEV *F*/*F*_0_) recorded over a 10‐s interval after the measurement of the maximum friction force in the ES clutch was calculated. The application of DC and AC voltages resulted in a normalized force standard value of approximately 0.07 in repeated tests, indicating the presence of a stick‐slip problem. The mechanisms of stick‐slip behavior under DC and AC voltages showed differences. For DC voltage, the maximum friction force was achieved, but external forces exceeding this threshold caused slipping, followed by a return to the stick phase due to the ES force of the ES clutch. In the case of AC voltage, the alternation of the ES force with voltage cycles caused stick‐slip behavior. The three‐phase ES clutch system alleviated this issue by sequentially engaging individual clutches, thereby decreasing the stick‐slip effect on the total force. This led to a normalized force standard value of 0.007. Therefore, the three‐phase voltage application reduces vibration by a factor of ten compared to DC and AC voltage applications, enabling more precise mechanical impedance control. The influence of residual charge may persist even after multiple test cycles. To verify the stability of the system, an additional 100 experiments were conducted on a single sample, and the results are presented in Figure S6 (Supporting Information). Under application of the three‐phase voltage, stable and consistent force values were maintained throughout all 100 cycles.

In conclusion, the three‐phase ES clutch demonstrated stable and consistent performance without force degradation and stick‐slip issues in repeated tests. These findings emphasize the ability of the three‐phase design to effectively address the limitations of conventional ES clutches, providing improved stability and reliability.

As the ES clutch does not move by itself, the friction response of the ES clutch was influenced by the speed of the external force applied. Higher speeds induced faster responses, but also intensified vibrations in the friction profile at excessive speeds as discussed in further detail in Figure S7 (Supporting Information). These vibration‐related issues were minimized by carefully matching the ES clutch frequency. To achieve precise force control, the frequency of the three‐phase voltage was adjusted. The frequency of the three‐phase voltage was regulated by adjusting the cycle of the square wave signal generated by the MCU and supplied to the three‐phase inverter. Experiments were performed by varying the frequency while maintaining a constant input voltage. The friction profile demonstrated different frequency‐dependent variations, as illustrated in Figure S8 (Supporting Information).

In conventional ES clutches utilizing AC voltage, a pronounced reduction in output force has been reported with increasing frequency.^[^ ^43^, ^46^ ^]^ However, due to the involvement of multiple interrelated factors, further investigation into the precise mechanisms underlying this frequency‐dependent force degradation remains challenging. In contrast, the three‐phase ES clutch exhibited stable force output even at higher frequencies, suggesting enhanced performance under dynamic operating conditions. At frequencies below 1 Hz, the three‐phase ES clutch exhibited behavior comparable to that of traditional ES clutches operating under DC voltage, characterized by intermittent motion and higher variability. However, at a frequency of 10 Hz, the clutch produced smoother motion with consistent and sustained force generation. Experimental results revealed that both the peak output force and the standard deviation decreased as the actuation frequency increased, underscoring the critical role of frequency tuning in optimizing ES clutch performance. Through iterative testing, 10 Hz was identified as the optimal operational frequency, yielding improved force stability and reduced fluctuation, which are essential for precise mechanical impedance control in soft robotic systems.

During operation of the ES clutch, periodic charging and discharging occur, requiring a small amount of power. The current was measured using a shunt resistor and the voltage was measured using a voltage divider. In the case of DC, energy is consumed once to charge a capacitor, resulting in 5.98 mJ. For AC, power is consumed only to the extent required to charge and discharge a single capacitor periodically. At 1 Hz, this results in a power consumption of 8.05 mW. The three‐phase voltage exhibits behavior similar to the AC case, with charging and discharging occurring sequentially across multiple layers. The higher measured power consumption of 76.2 mW at 10 Hz and 400 V primarily results from the higher operating frequency compared to the 1 Hz used in the AC case.

The power consumption of an ES clutch is theoretically the product of the switching frequency and the energy required to charge the capacitor:^[^ ^35^ ^]^

(2) $$P=\frac{\varepsilon_{0}\varepsilon_{r}AV^{2}f}{d}$$

where *P* is the power, *d* the insulator thickness, *V* the actuation voltage, and *f* the frequency. For an ES clutch with a 5 µm‐thick polyamide layer of relative permittivity 3.4, actuated at 10 Hz and 400 V, the calculated power consumption is 41.8 mW. The experimentally measured value of 76.2 mW indicates additional losses beyond the ideal model.

### 2.4 Mechanical Impedance Control

#### 2.4.1 Force Control System

When a consistent amplitude of three‐phase voltage is applied to the 3‐phase ES clutch, a constant friction is maintained. However, as the length of the three‐phase ES clutch increases, the contact area decreases, leading to a reduction in friction. To prevent this reduction, the applied voltage must be adjusted to compensate for the change in length. This adjustment is achieved through the implementation of an open‐loop control system.

**Figure 5:**
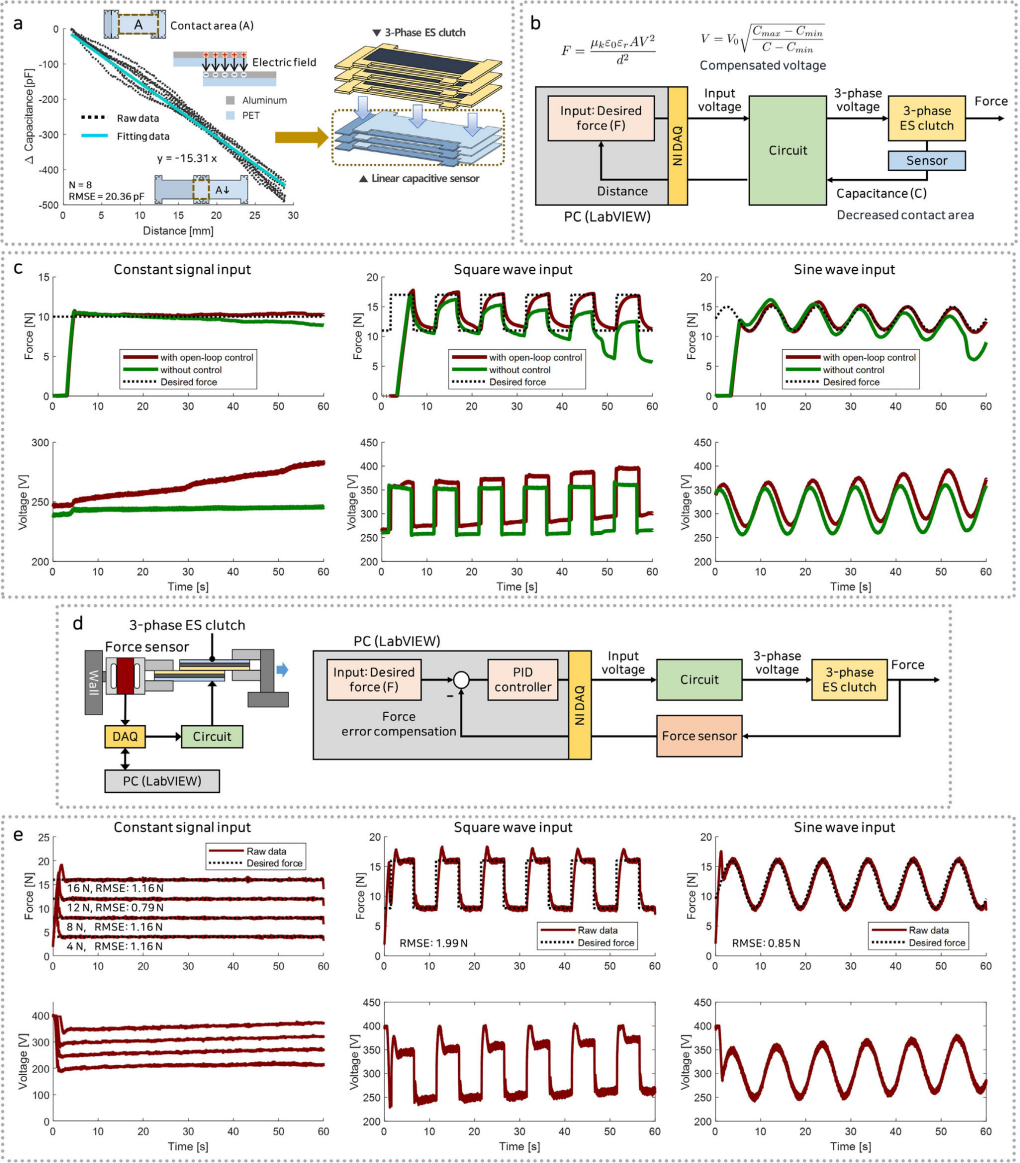
Three‐phase ES clutch control system for compensating the decreased contact area. a) Three‐phase ES clutch with a linear capacitive sensor for measuring the contact area. b) Open‐loop control system with the linear capacitive sensor. c) Open‐loop control friction results with constant, square, and sine wave input signals. d) Three‐phase ES clutch PID feedback control system with a force sensor. e) Three‐phase friction feedback control results with constant, square, and sine wave input signals.

A linear capacitive sensor was developed and integrated into the three‐phase ES clutch module to measure length variations (**Figure** **5**a). The sensor was fabricated by laser cutting an aluminum‐deposited PET film to match the dimensions of the three‐phase ES clutch, forming a three‐layered structure. The capacitance of the sensor changes proportionally with the contact area, and an resistance‐capacitance (RC) low‐pass filter embedded in the circuit processes these signals, enabling length measurement. This film‐based variable capacitive sensor detects changes in capacitance caused by variations in contact area, which are manifested as reductions in the amplitude of the AC signal. Sensor performance was evaluated through a pull‐in test conducted under frictional conditions on the three‐phase ES clutch. Results demonstrated that a displacement of 25 mm corresponded to a capacitance change of approximately 400 pF, with the sensor showing exceptional linearity and an RMSE of 20.36 pF. By providing real‐time length measurements, the sensor enables the application of a compensated voltage to account for elongation, ensuring that the three‐phase ES clutch consistently maintains stable friction performance under varying length conditions.

An open‐loop control system was developed to maintain consistent friction by applying compensated voltage, as depicted in Figure 5b. The circuit processed the input signal and converted it into a three‐phase voltage, with the amplitude directly proportional to the voltage applied to the three‐phase ES clutch. To measure capacitance, a 1500 Hz square wave AC signal was applied to the sensor. Changes in the length of the three‐phase ES clutch altered the sensor's capacitance, which was reflected as variations in the amplitude of the return signal. The processed signal was transmitted to a DAQ device, where capacitance values were measured and analyzed via a LabVIEW program. The program calculated the required compensation based on detected length variations and adjusted the voltage in real time to achieve the desired force. The computed voltage signal was then input into the circuit through the DAQ device. The derivation of the compensated voltage model is provided in the Note S1 (Supporting Information). The governing equations are expressed as follows:

(3) $$\begin{array}{cc} & V=V_{0}\sqrt{\frac{C_{max}-C_{min}}{C-C_{min}}}\\ & V_{0}=\sqrt{\frac{F_{target}d^{2}}{\mu_{k}\varepsilon_{0}\varepsilon_{r}A}} \end{array}$$

where *C* represents the sensor capacitance, and *C*_*max*_ and *C*_*min*_ denote the initial maximum and minimum capacitance values, respectively. The parameter *V*_0_ was determined using the target force *F*_*target*_. By accurately measuring the length of the three‐phase ES clutch in real time, the sensor enables the application of a compensated voltage to effectively account for elongation. This capability ensures that the three‐phase ES clutch consistently maintains its frictional performance, even under varying operational conditions.

The effectiveness of friction control in the proposed three‐phase ES clutch was evaluated through experiments conducted under two conditions (Figure 5c: without control and with an open‐loop control system utilizing a linear capacitive sensor. Pull‐in tests were performed while maintaining a constant target force to evaluate the system's ability to ensure stable force levels. In the uncontrolled system, friction progressively decreased during the test, reflecting its inability to maintain consistent performance over time. In contrast, the open‐loop control system dynamically adjusted the applied voltage based on length variations detected by the linear capacitive sensor, maintaining consistent friction throughout the test. To further evaluate the system's adaptability under dynamic conditions, sinusoidal and square‐wave signals with a frequency of 0.1 Hz were applied to test its ability to track variable target forces. The uncontrolled system exhibited a gradual decrease in friction during repetitive wave inputs, demonstrating its limitations in compensating for variations in the target force. In contrast, the open‐loop control system accurately followed the input waveforms, maintaining stable friction, and demonstrating its effectiveness under dynamic conditions.

A force feedback control system utilizing a force sensor was implemented to improve the precision of the three‐phase ES clutch (Figure 5d). A custom tensile test setup equipped with a force sensor was employed to accurately measure and monitor force values. These measurements were collected via a DAQ and analyzed using a LabVIEW program integrated with a PID (Proportional‐Integral‐Derivative) control system. The PID system calculated the error between the actual and target forces and made real‐time voltage adjustments to ensure precise force control. PID gain values were optimized through iterative testing and the compensated voltage was applied to the three‐phase ES clutch via the control circuit. Pull‐in tests using constant, square and sinusoidal signals (Figure 5e) were performed to evaluate the effectiveness of the PID system. The PID feedback control system continuously monitored friction, promptly corrected detected errors, and accurately tracked target force values. Although reductions in the contact area within the three‐phase ES clutch led to decreases in force, the force sensor immediately detected these changes, allowing real‐time voltage adjustments to maintain consistent force control. Therefore, the RMSE values obtained from the three test cases were measured as 1.16 N (constant), 1.99 N (square), and 0.85 N (sinusoidal), respectively, which verified the improved control performance. Voltage overshoot may occur during testing, indicating the need for PID calibration and parameter optimization to prevent the maximum voltage from exceeding the dielectric breakdown threshold, despite the improved responsiveness of the system.

#### 2.4.2 Mechanical Impedance Control

**Figure 6:**
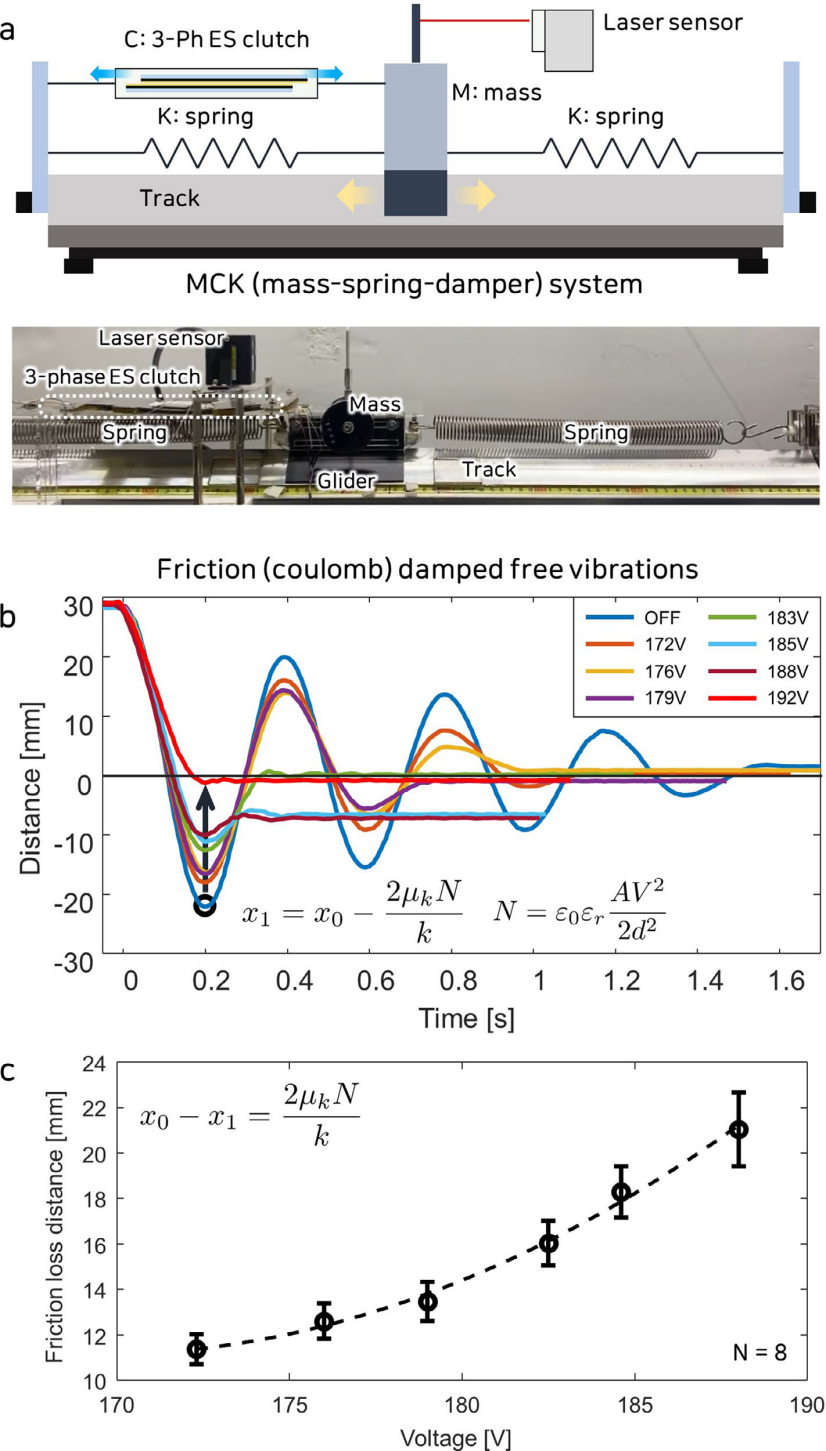
A damping system with a three‐phase ES clutch serving as a damper. a) MCK oscillation system experimental setup. b) Results showing friction‐damped vibrations with the ES clutch damping effect. c) Friction loss distance as a function of applied voltage.

To validate the capability of the three‐phase ES clutch to provide variable mechanical impedance, an experiment utilizing an MCK vibration system was designed. The experimental setup, shown in **Figure** **6**a, included a spring, glider, linear air track, actuator jig frame, and a distance laser sensor. This study investigated how the damping effect in an MCK system varies with applied voltage, using the three‐phase ES clutch as a damper. The experimental apparatus used air supplied by an external pump, directed through small holes in the linear air track to form an air cushion. This configuration effectively prevented direct contact between the glider and the track surface, significantly reducing friction. The glider was equipped with masses and springs to adjust the resonance frequency. Both the springs and the three‐phase ES clutch were fixed to designated positions on the actuator jig frame to induce controlled oscillations. The spring constant and mass were carefully calibrated to achieve the desired oscillation speed and displacement range. To minimize the effect of the inherent initial friction of the ES clutch on the test results, a high spring constant was selected. The system was configured to produce approximately three oscillations in the absence of a damping force. A laser distance sensor measured the displacement of the glider, enabling the analysis of damping effect characteristics. When the glider was released from a position 30 mm away from its neutral point, it began to oscillate due to the spring force. The displacement of the glider was recorded throughout the MCK system's oscillations. The oscillation period remained constant at approximately 0.2 s, regardless of the damping force, indicating the stability of the system and consistent behavior under varying damping conditions. As the glider's position converged, it stopped at the equilibrium point determined by the spring constant. The initial displacement peak, measured around 0.2 s, was used to calculate the damping effect. This was done using the Coulomb friction model integrated into the MCK system. The damping effect was quantified by analyzing the attenuation of displacement caused by friction as a function of the applied voltage. The displacement variation at the first peak (*x*_1_) was calculated using the Coulomb friction model, which relates damping force to system parameters, expressed as:

(4) $$x_{1}=x_{0}-\frac{2\mu_{k}N}{k}$$

where *x*_0_ is the initial displacement, µ_*k*_ is the kinetic coefficient of friction, *N* is the normal force of the ES clutch, and *k* is the spring constant.

Figure 6b illustrates that an increase in applied voltage significantly enhances the damping force, as evidenced by a notable reduction in the amplitude of the displacement peaks. This finding emphasizes the ability of the system to perform precise damping control. The damping effect was indirectly evaluated by calculating the friction loss distance (*x*_0_ − *x*_1_) at the displacement peaks. The friction loss distance was derived from the displacement data, shown in Figure 6c, to examine the relationship between the damping effect and the applied voltage. Due to the influence of initial conditions, including inherent friction within the system, the response of the model may be affected. Therefore, experiments were conducted to capture the overall behavioral trends of the model. The results, represented through a second‐order polynomial fitting, were consistent with the trends predicted by the maxwell pressure model.

#### 2.4.3 Application to the Wearable System

In wearable systems, variable impedance control is required to assist eccentric and isometric contractions. Previous studies utilizing ES clutches have primarily focused on supporting isometric contractions,^[^ ^33^, ^57^, ^58^ ^]^ as conventional ES clutches are unable to function under dynamic conditions. However, eccentric contractions are more relevant to daily activities, such as squatting or descending stairs, where muscles lengthen while generating force. In this study, we demonstrate that the three‐phase ES clutch can regulate variable impedance to support eccentric contractions under dynamic conditions. Such a system can be applied either to reduce muscle load for assistive purposes or to increase muscle effort by applying controlled external resistance, enabling use in rehabilitation application.

**Figure 7:**
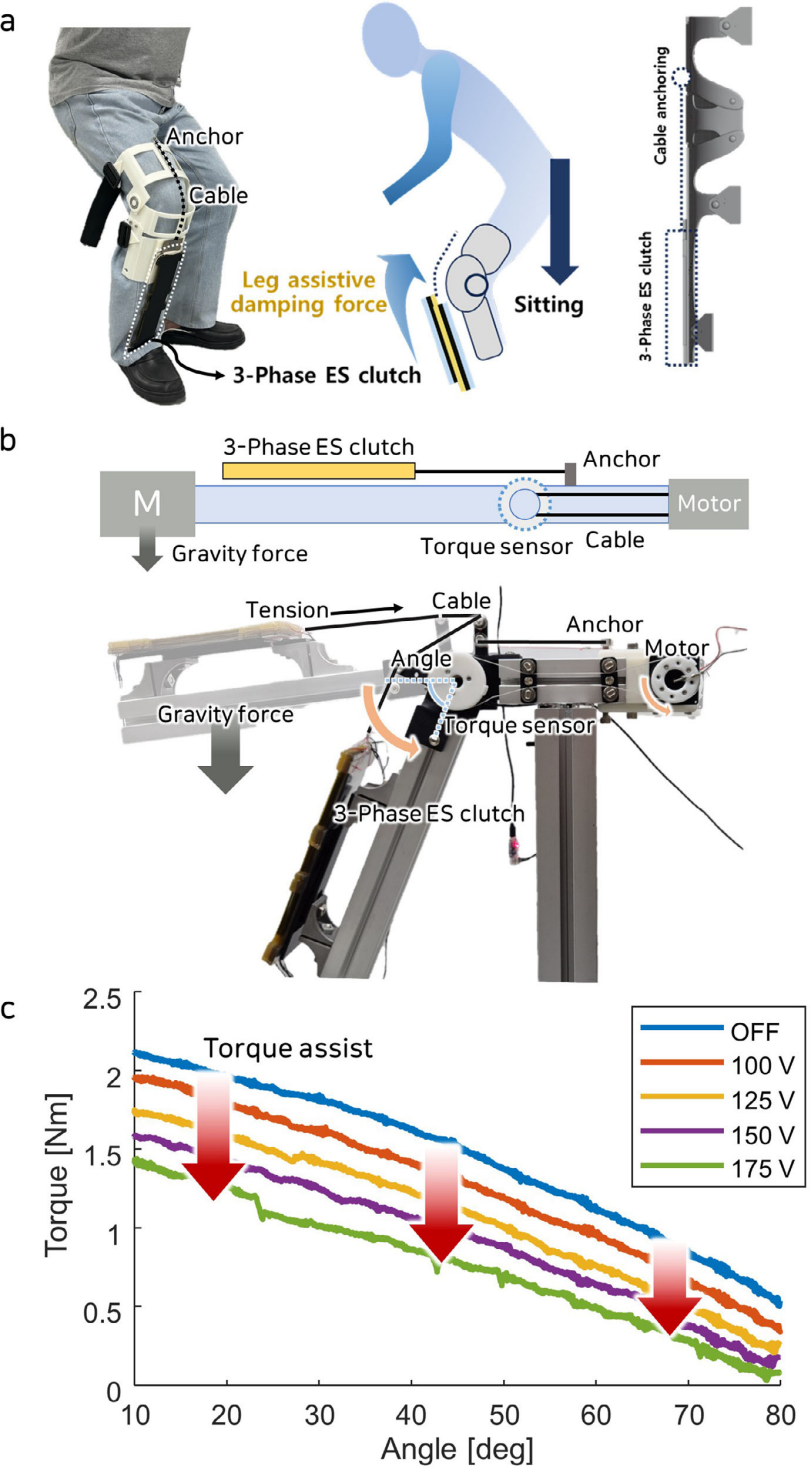
Three‐phase ES clutch application for the wearable system. a) Wearable leg assist system with the Three‐phase ES clutch and assist mechanism. b) Experimental setup for measuring damping force to assist joint torque. c) Damping force as a function of joint angle and applied voltage.

To validate this approach, the three‐phase ES clutch was integrated into a wearable system and employed as a damper to assist eccentric contraction movements, which are critical for muscle strengthening and rehabilitation. Squatting was selected as the representative activity for eccentric exercise. As shown in **Figure** **7**a, the wearable system was designed to support thigh muscle activity during squat motions, thereby enhancing lower‐limb stability and reducing muscular strain during repetitive movements. The system transmits resistance through a structure composed of anchors affixed to the thigh and shin, a knee brace, and connecting cables. To increase the level of muscle assistance, an additional layer of the three‐phase ES clutch was incorporated into the system.

An experiment was conducted, as illustrated in Figure 7b, to evaluate the effectiveness of the three‐phase ES clutch in reducing joint torque via its damping mechanism. The experimental setup comprised two frames connected by a joint, a cable, a torque sensor, a motor, and the three‐phase ES clutch. A torque sensor was installed on the joint to measure torque values, while a motor connected to the joint through a cable was used to adjust the joint angle. The ES clutch provided variable friction by altering its length in response to changes in the joint angle. The ES clutch's cable passed through the joint and was anchored to the upper frame, ensuring stable force transmission and consistent friction adjustment throughout the experiment. Bearings were incorporated into the joint to facilitate flexible fixation relative to the joint angle. The motor regulated the joint angle, and the experiment aimed to record variations in torque as influenced by the frictional force from the ES clutch. As the joint angle increased, the gravitational moment acting on the frame decreased, leading to a reduction in joint torque. The ES clutch dynamically adjusted its friction in accordance with the joint angle, thereby assisting in torque modulation.

Figure 7c depicts the relationship between torque values and joint angles, offering insights into how the damping effect of the ES clutch varies with joint movement. The joint angle was consistently adjusted within a range of 10 to 80 degrees at a speed of 0.5 degs^−1^. Throughout this process, a fixed voltage was applied to the ES clutch, generating a damping effect. The applied voltage was incrementally varied from 100 to 175 V in steps of 25 V, remaining below the maximum allowable limit to avoid motion interference. The results demonstrate the ability of the ES clutch to enable precise control. As the applied voltage increased, greater assistance was provided, resulting in reduced torque values across all joint angles. In the absence of frictional assistance from the ES clutch, a torque value of 2.2 Nm was recorded at a joint angle of ten degrees, primarily caused by the weight of the frame and fixed parts in the experimental setup. When 175 V was applied to the ES clutch, the torque at 10 degrees decreased to 1.4 Nm, indicating a reduction of 0.8 Nm due to the damping effect. This corresponds to a 36% reduction in the torque load supported by the three‐phase ES clutch, underscoring its potential to significantly alleviate joint strain in wearable systems. Through the damping effect of the three‐phase ES clutch, torque assistance can be achieved during joint movements in wearable systems.

## 3 Discussion and Conclusion

This study investigates the development and implementation of a three‐phase ES clutch to address challenges associated with residual charge and force degradation in conventional ES clutch systems. The proposed three‐phase ES clutch is specifically designed to reduce the effects of residual charge and improve operational control through the application of a three‐phase voltage modulation method. The system demonstrates enhanced performance and stability compared to traditional ES clutches, consistently maintaining reliable functionality. By employing a phase modulation technique, the three‐phase ES clutch effectively eliminated the effects of residual charge and minimized stick‐slip issues. Furthermore, the integration of a linear capacitive sensor and a force sensor enabled precise control over the frictional force generated by the clutch. The MCK experiment confirmed the effectiveness of the variable mechanical impedance control system in efficiently modulating mechanical impedance. Additionally, the clutch's potential to support muscle eccentric contractions was validated through its incorporation into a wearable system.

The three‐phase ES clutch demonstrates significant potential for precise control, but key challenges remain. The force generated by the clutch is influenced by the speed at which the force is applied, the frequency of the applied signal, and the mechanical properties of the dielectric layer. Matching the frequency of the applied signal to the pulling speed is essential for maintaining stability and minimizing force fluctuations. Since the optimal frequency varies with speed, a systematic approach is required to enable effective frequency adaptation under varying speed conditions. This strategy can suppress vibration and improve operational consistency. In addition, the mechanical properties of the materials and the thickness of the dielectric layer are critical, as excessive shear forces can lead to stick‐slip phenomena. Materials with higher stiffness reduce deformation, enabling faster and more stable responses under dynamic conditions. Therefore, optimizing performance requires simultaneous tuning of the pulling speed, applied frequency, and material properties. These interdependent parameters necessitate an integrated approach to ensure overall efficiency and reliability. By dynamically aligning signal frequency with the speed by applied force and utilizing advanced dielectric materials, the ES clutch can achieve enhanced control precision.

A basic experiment was conducted to evaluate the applicability of the three‐phase ES clutch in a wearable system. Although the results demonstrated basic feasibility, more research is required to improve the effectiveness of the system. Enhancing overall performance will require optimization of both the mechanical design and control strategies. Furthermore, a quantitative evaluation of the muscle load under mechanical impedance control is necessary to verify the assistive functionality of the system. This will be addressed in future work. Additional validation is also planned for various applications, including posture support and rehabilitation devices. Future studies will target dynamic movements requiring variable impedance, such as stair descent and sit‐to‐stand transitions. Improved adaptability is expected to enhance assistive performance and expand the applicability of the system in rehabilitation scenarios.

## 4 Experimental Section

### Fabrication process

The ES clutch consisted of three main layers: a protective layer, an electrode, and a dielectric layer. The protective layer uses a hand coating film (100 µ*m* thickness, PET), cut twice using a CO2 laser cutting machine (Coryart) with adjustable intensity: first cutting the release film and then cutting the outline. For the electrode, a conductive carbon tape (45 µ*m* thick, from Woo Yang Korea Chemical) was attached to the area where the release film was removed from the hand‐coating film. A copper wire electrode was connected to the carbon tape to enable connection to a circuit. The remaining release film was peeled off the hand coating film. The dielectric layer was a PI film (5 µ*m* thickness, polyimide, Isoflex KESPI), which was fixed onto an acrylic plate using PET tape under consistent tension to avoid wrinkles. The prepared hand coating film layer was attached to the PI film, ensuring that no bubbles were trapped between the two films by using a roller. The outline of the PI film was cut using a utility knife. Each single layer ES clutch was manufactured with an area of 8 cm × 15 cm, a thickness of 150 µ*m*, and a weight of 3 g. If the ES clutch was warped due to film tension, it was placed between flat plates, compressed, and allowed to settle flat over time. Six units were prepared for the three‐phase clutch. For a multilayer configuration, an adhesive film was used to fix the layers. A thick adhesive film was required to secure space between the each films. To increase the thickness of the adhesive film, two double‐sided acrylic adhesive sheets were attached to both sides of a 100 µ*m* PC (polycarbonate) sheet. To prevent bubbles during the adhesion process, a spray of water was applied between the two surfaces and a roller was used to press it evenly. The manufactured adhesive layer was cut into the desired outline using a laser cutter. ES clutches were aligned, set, and fixed with parts of the adhesive layer between them to produce a three‐phase ES clutch. To measure the reduction in contact area caused by the length variation in the three‐phase ES clutch, a linear capacitive sensor was developed and integrated. The sensor consists of a 100 µ*m* aluminum‐deposited PET film (PET dielectric constant; 3.0), which was laser cut to match the dimensions of the ES clutch. Three sets of capacitive sensor films were stacked to form a multilayer structure, thereby increasing the total capacitance. Acrylic‐based double‐sided adhesive sheet were inserted between the sensor layers to bond them and PC spacers were used to maintain small gaps. This multilayer configuration ensures stable and repeatable capacitive variation in response to distance change. The completed sensor was then attached to the three‐phase ES clutch using the acrylic adhesive sheet. The fabricated actuator exhibits dimensions of 80 mm by 200 mm by 6 mm, with a mass of 50 g.

### Experiment setting

1) Tensile test: To verify the friction performance of the three‐phase ES clutch, a tensile test was performed. A force sensor (Futek, LSB302) and a fixed part were placed on a three‐axis CNC machine. The ES clutch was connected to the force sensor and the fixed part. The CNC machine pulled the ES clutch along the x‐axis. In this experiment, it was pulled at a specific speed. The force sensor measured the friction of the ES clutch, and the signal was amplified using a dedicated amplifier (Futek, IAA100). The friction value was transmitted as an analog signal to a DAQ (NI USB‐6212). The data calculated by a LabVIEW program on a PC was stored at a sampling rate of 1 kHz. Various tests were conducted on the same ES clutch by adjusting the voltage. A high voltage DC / DC converter (EMCO, Q05‐12) was used to adjust the applied voltage on the ES clutch. To apply AC voltage and three‐phase voltage to the ES clutch, a custom switching circuit was used to control the applied voltage.
(2) Measurement of power consumption: To evaluate the power consumption of the three‐phase ES clutch, simultaneous current and voltage measurements were conducted. A 3 Ω shunt resistor was placed at the GND of the high voltage DC/DC converter, and the current flowing through the resistor was measured using a current sensing amplifier (TI, INA282‐Q1). For voltage measurement, a voltage divider consisting of 10 MΩ and 100 kΩ resistors was used to reduce the high voltage to a measurable range. While the ES clutch was operating under load, current and voltage signals were transmitted as analog inputs to a data acquisition device (NI USB‐6212). The power consumption was then calculated and recorded in real time using a LabVIEW program on a PC, with a sampling rate of 1 kHz.
3) Damping effect test: The three‐phase ES clutch acted as a damper due to friction, demonstrating a damping vibration effect in the experiment. A glide was placed on an air track and the mass and spring were connected to enable adjustment and analysis of the reciprocating vibration characteristics. Additional jigs for securing the ES clutch and spring were made by laser cutting an acrylic plate. A custom circuit was used to apply the three‐phase voltage to the ES clutch. A CMOS laser sensor (Omron, ZX1‐LD300A61) was placed on the air track to measure the glider displacement, and data were collected using DAQ (NI USB‐6212). Considering the measurement range of the laser sensor, the initial position of the glider was set to 3 cm. The laser sensor was fixed using an acrylic plate that was made by laser cutting. The data calculated by a LabVIEW program on a PC was stored at a sampling rate of 1 kHz.
4) Wearable system: An experiment was conducted to verify the torque assistance provided by the damping effect in a wearable application of the three‐phase ES clutch. To quantify torque assistance, a frame structure including a 1DOF joint and a torque sensor (ATI, F/T mini45) were used to measure torque. Bearings and torque sensors were attached to the joints and the connecting parts between each frame were made using a 3D printer (Stratasys, ABS‐P430). The motor (Robotis, H54‐200‐S500) was connected to the joint by a cable and the joint angle was adjusted by inputting the angle to the motor. The motor was controlled using code written in LabVIEW. The three‐phase ES clutch was fixed to the frame and assistive torque was generated as the three‐phase voltage was applied. The torque values corresponding to the joint angles were collected by the torque sensor using a DAQ (NI USB‐6212). The signal was stored at a sampling rate of 1 kHz using a LabVIEW program. The prototype of the wearable system was manufactured using a 3D printer. Rubber bands were used to hold the wearable parts on the shin and thigh. To ensure movement of the knee joint, a joint structure was implemented using bearings. The ES clutch was placed on the shin, connected by a cable that extended to the anchor part of the thigh. A cable guide was used to ensure low‐friction movement of the cable. As the ES clutch operated, assistive torque was generated at the joint.

### Circuit

To apply a three‐phase voltage to the three‐phase ES clutch, a custom circuit was developed. The target voltage was set using a LabVIEW program, and the analog signal was transmitted to the circuit through a DAQ (NI USB‐6212). A buffer (LM7171BIN) was used to prevent voltage drop. A high voltage DC / DC converter (EMCO, Q04‐12) boosted 12 to 500V. To control the high‐voltage signal in three‐phase switching, a MCU (Arduino Nano), three gate drivers (IR2101PBF), and six IGBTs (IRG4BC20KDPBF) were used. The repeated three‐phase switching signals were generated by the MCU and sent to the gate drivers. Gate drivers were used to control the voltage switching on the IGBTs. The IGBTs could switch between HV and GND, applying a three‐phase voltage to the three‐phase ES clutch. To verify the applied voltage level, a voltage divider circuit with a ratio of 1/100 was implemented. The voltage signal was collected through a DAQ. The voltage applied to the ES clutch was calculated and stored at a sampling rate of 1 kHz using a LabVIEW program. Additionally, the MCU generated a 1 kHz square wave signal, which was sent to the linear capacitive sensor. The capacitance value changed depending on the contact area, causing a change in the response of the square wave. The analog signal was collected through the DAQ. The amplitude of the analog signal was analyzed using a LabVIEW program, and the capacitance value was calculated and stored at a sampling rate of 100 Hz.

## Conflict of Interest

The authors declare no conflict of interest.

## Author Contributions

D.L. and J.B. conceived and designed the experiments. D.L. and H.Y. fabricated the device. D.L. and H.Y. performed experiments. D.L. and J.B. analyzed the data and discussed the results. D.L. and J.B. contributed to the writing of the manuscript.

## Supporting information

Supporting Information

## Data Availability

The data that support the findings of this study are available from the corresponding author upon reasonable request.
