# Supplementary material for: A Three‐Phase Electrostatic Clutch with Variable Mechanical Impedance Control for Soft Robotic Systems
Source: Adv Sci (Weinh). 2025 Sep 16;12(45):e10291. doi: 10.1002/advs.202510291 (PMC12677595; doi:10.1002/advs.202510291)
Supplement: Supplementary file 1 — Supporting Information [file ADVS-12-e10291-s001.pdf]

Supporting Information:

A 3-Phase Electrostatic Clutch with Variable Mechanical Impedance Control for Soft Robotic Systems

Dongyoung Lee, Heejin Yu, Joonbum Bae*

School of Mechanical Engineering, Korea University, Seoul, Korea
*Email Address: jbbae@korea.ac.kr

Supporting Information

In this supplementary document, we provide (Note S1) the calculation of linear capacitance sensor, (Figures S1 to S5) the signalling process of ES clutch, (Figures S6 to S8) the characteristics of ES clutch, and (Table S1) the performance parameters of ES clutch.

Note S1: Linear capacitance sensor and compensated voltage modeling and for open loop control

As shown in Figure 5b, we implemented the linear capacitance sensor and compensated voltage modeling for open-loop control in the ES clutch. ES clutch force (F) follows the Maxwell pressure model.

$$F = \frac{\varepsilon_0 \varepsilon_r A V^2}{d^2} \tag{S1}$$

F varies with the contact area (A) and voltage (V).

$$F_0 = F_1 \tag{S2}$$

F should maintain a constant force during the tensile test.

$$\begin{aligned} \frac{\varepsilon_0 \varepsilon_r A_0 V_0^2}{d^2} &= \frac{\varepsilon_0 \varepsilon_r A_1 V_1^2}{d^2}, \\ V_1 &= V_0 \sqrt{\frac{A_0}{A_1}} \end{aligned} \tag{S3}$$

Given A_0 and V_0 as initial values, if A_1 is reduced due to a change in length, and if V_1 is applied to compensate for this, the equation for V_1 is organized in terms of A.

$$\begin{aligned} C &= \frac{\varepsilon_0 \varepsilon_r A}{d}, \\ V &= V_0 \sqrt{\frac{C_{max} - C_{min}}{C - C_{min}}} \end{aligned} \tag{S4}$$

A linear capacitance sensor is capable of determining the capacitance (C) and subsequently computing the contact area. By expressing A as a function of C, an equation for V can be derived.

$$V_0 = \sqrt{\frac{F_0 d^2}{\varepsilon_0 \varepsilon_r A}} \tag{S5}$$

V_0 can be calculated via the initial target force F_0.

Supplementary Figures

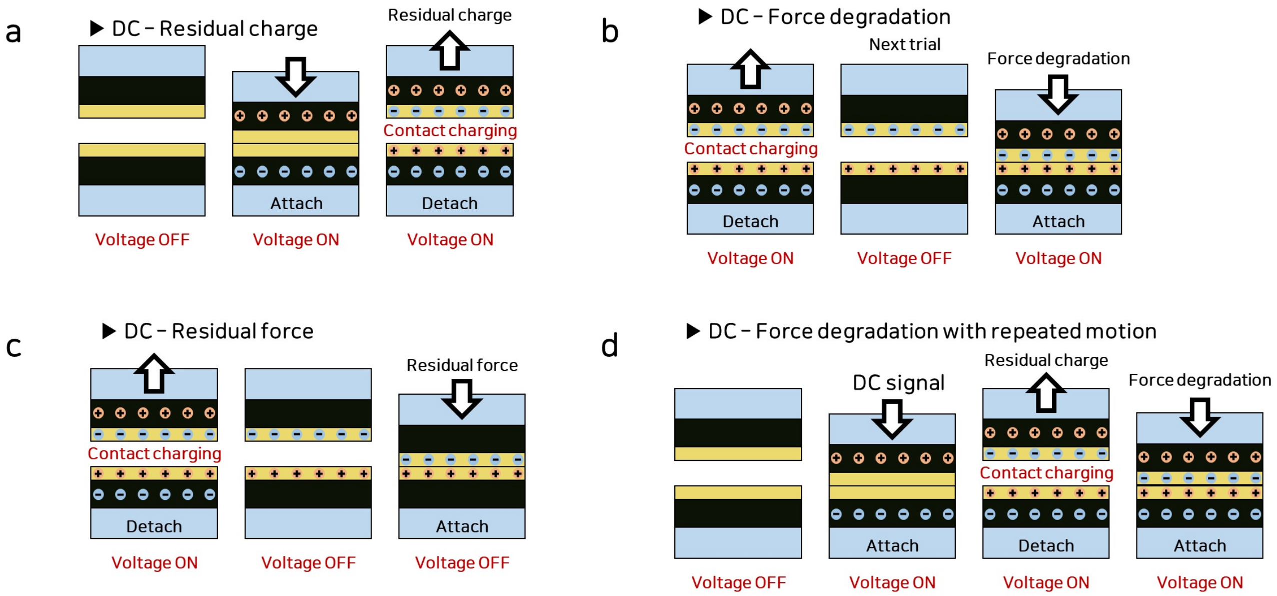

Figure S1: **Contact charging mechanism with DC voltage application.** (a) Residual charge issue. ES clutch generate residual charge when detached while voltage ON state. (b) Force degradation issue. ES clutch show reduced electrostatic force due to existing residual charge which caused force degradation issue. (c) Residual force issue. ES clutch show unexpected interact force due to the residual charge while voltage OFF state. (d) Force degradation with repeated motion. ES clutch accumulate the residual charge for repeated test which cause the amplify force degradation issue.

The non-linear characteristics observed during the operation of the ES clutch can be attributed to the presence of residual charges. Existing research has established that these residual charges typically originate from leakage currents. However, the influence of these charges is often mitigated as a result of the substantial time constants involved. Experimental evidence has indicated that the occurrence of residual charges is predominantly due to the phenomenon of contact charging. This phenomenon becomes more pronounced when an external electric field is applied, facilitating easier charge mobility during contact. Under normal circumstances, identical materials tend to have similar energy levels, which prevents charge transfer. However, alteration of energy levels in the presence of an external electric field creates conditions more favorable for contact charging.

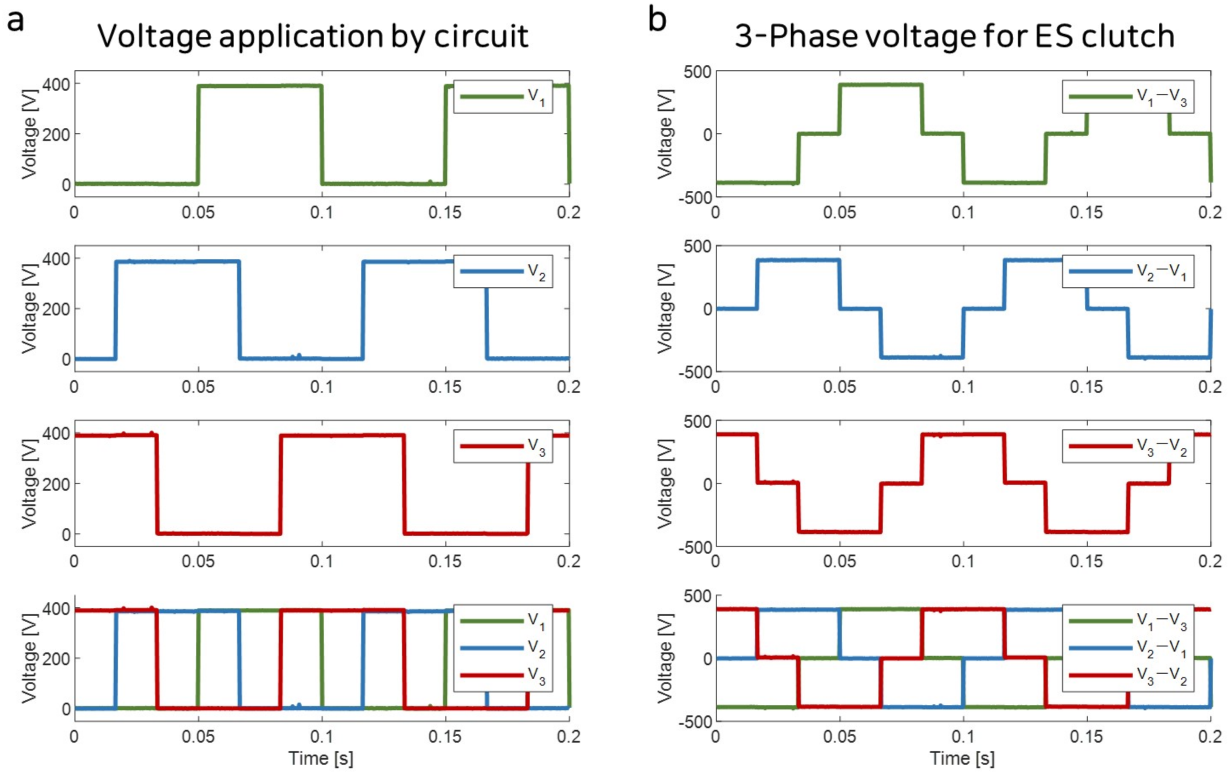

Figure S2: **Activation process of the 3-phase ES clutch in real time.** (a) Circuit-level voltage application process. (b) 3-phase voltage waveform applied to the ES clutch.

To analyze the actuation process, the voltages applied to both the control circuit and the 3-phase ES clutch were measured. The three input signals, V_1, V_2, and V_3, exhibit a 120° phase difference relative to each other. Each voltage signal operates at 10 Hz, resulting in a period of 0.1 seconds per cycle. The "3-phase voltage for ES clutch" represents the actual voltages applied to each individual electrode of the clutch, illustrating the differential voltages responsible for generating electrostatic forces. The full actuation cycle consists of six sequential steps, corresponding to a total period of 0.1 seconds.

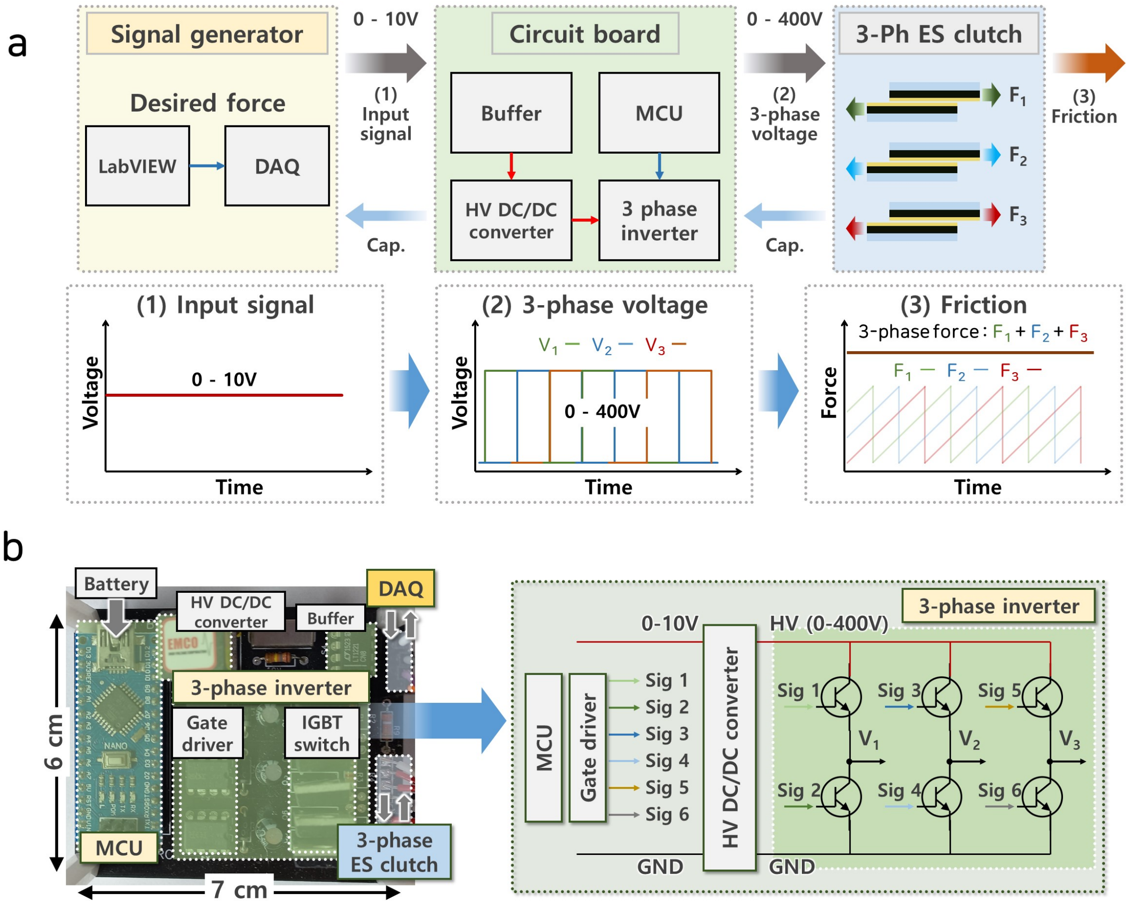

Figure S3: **Signaling process for 3-phase voltage.** (a) Overall signal process with the signal generator, the circuit board, and the 3-phase ES clutch. (b) Circuit for the 3-phase ES clutch that includes a 3-phase inverter to generate 3-phase voltage.

As illustrated in Figure S3a, the proposed system comprises three primary components: a signal generator, a circuit board, and a 3-phase ES clutch. The signal processing begins with the signal generator producing an input signal, which is transmitted to the circuit board. The circuit board then converts this input into a 3-phase signal to activate the ES clutch, generating a continuous friction profile. Within the signal generator, LabVIEW program with a Data Acquisition (DAQ) device, calculates the voltage required to achieve the target force. This voltage signal is subsequently transmitted to the circuit board for further processing. A customized circuit functions as a 3-phase inverter to produce the 3-phase voltage, as shown in Figure S3b. This circuit can modulate the friction with voltage regulation. Detailed specifications of circuit setup are provided in Figure S4. The 3-phase high-voltage system incorporates several key components: a high-voltage converter, a microcontroller unit (MCU) for 3-phase switching control, insulated gate bipolar transistors (IGBTs) as switches, and gate drivers to manage the IGBT signals. The high-voltage converter amplifies input voltages ranging from 1–5 V to a maximum of 400 V, ensuring linear output amplification across this range. For single-electrode operation of the ES clutch using a square wave signal, two switches are employed. The node between these switches connects to the ES clutch electrode, alternating the electrode between HV and GND based on the ON/OFF states of the switches. Gate drivers control the IGBT switches to enable this operation. To apply a 3-phase voltage, six switches are arranged to supply voltage to the three electrodes. The MCU sends six discrete signals through the gate drivers, activating the switches in the inverter circuit. This configuration ensures precise phase modulation of the applied voltage, resulting in a 3-phase voltage at the ES clutch electrodes. Each electrode receives a distinct phase voltage, creating force within its respective layer of the ES clutch. The interaction of these forces produces a continuous output force.

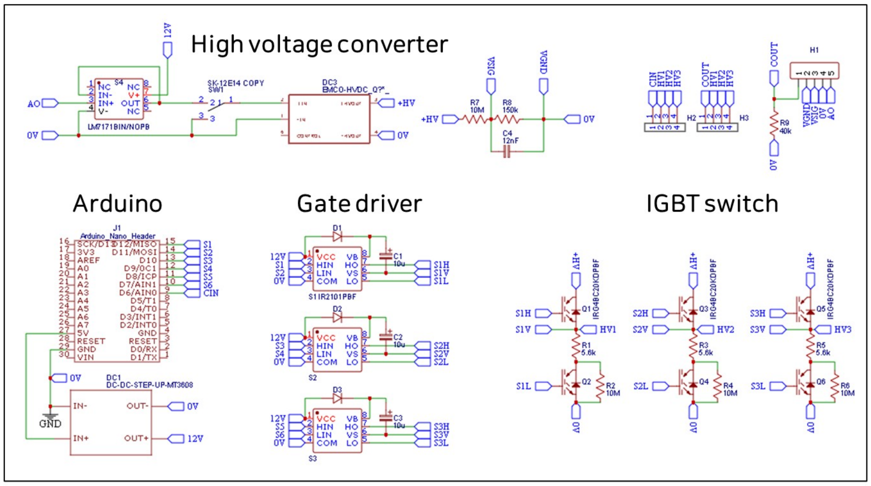

Name	Designator
10u	C1,C2,C3
12nF	C4
1N4007	D1,D2,D3
DC-DC-STEP-UP-MT3608	DC1
EMCO-HVDC_Q04-5	DC3
HDR-F-2.54_1x5	H1
4 Pin Female Header	H2,H3
Arduino_Nano_Header	J1
IRG4BC20KDPBF	Q1,Q2,Q3,Q4,Q5,Q6
5.6k	R1,R3,R5
10M	R2,R4,R6,R7
150k	R8
40k	R9
IR2101PBF	S1,S2,S3
LM7171BIN/NOPB	S4
SK-12E14 COPY	SW1

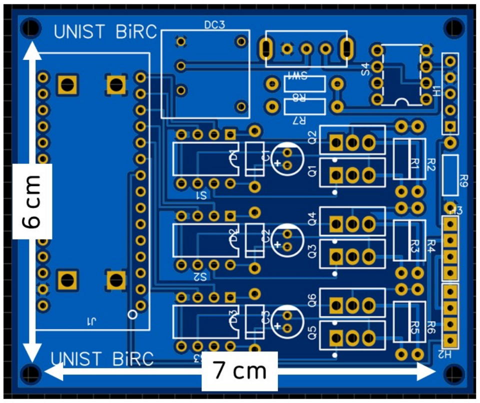

Figure S4: **Schematic circuit for 3-phase voltage application with 3-phase inverter and high voltage converter.**

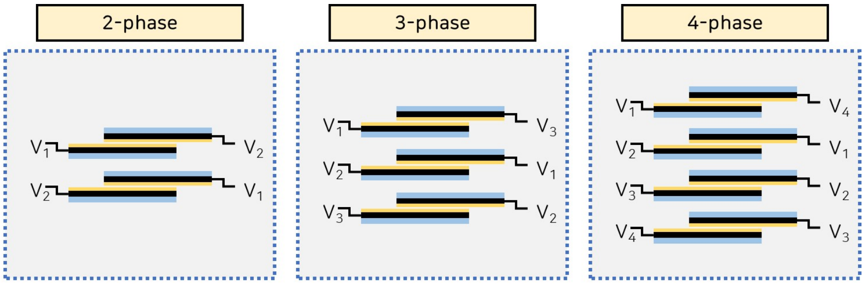

▶ Multi-phase ES clutch activation process

Step	V_1	V_2	$V_1 - V_2$	$V_2 - V_1$	Force
1	1	0	-1	-1	$2F_0$
2	1	1	0	0	0
3	0	1	1	1	$2F_0$
4	0	0	0	0	0

▶ 2-phase voltage application

Step	V_1	V_2	V_3	$V_1 - V_3$	$V_2 - V_1$	$V_3 - V_2$	Force
1	1	0	0	1	-1	0	$2F_0$
2	1	1	0	1	0	-1	$2F_0$
3	0	1	0	0	1	-1	$2F_0$
4	0	1	1	-1	1	0	$2F_0$
5	0	0	1	-1	0	1	$2F_0$
6	1	0	1	0	-1	1	$2F_0$

▶ 3-phase voltage application

Step	V_1	V_2	V_3	V_4	$V_1 - V_4$	$V_2 - V_1$	$V_3 - V_2$	$V_4 - V_3$	Force
1	1	1	0	0	1	0	-1	0	$2F_0$
2	1	1	1	0	1	0	0	-1	$2F_0$
3	0	1	1	0	0	1	0	-1	$2F_0$
4	0	1	1	1	-1	1	0	0	$2F_0$
5	0	0	1	1	-1	0	1	0	$2F_0$
6	1	0	1	1	0	-1	1	0	$2F_0$
7	1	0	0	1	0	-1	0	1	$2F_0$
8	1	1	0	1	0	0	-1	1	$2F_0$

▶ 4-phase voltage application

Figure S5: **Multi-phase ES clutch.**

A separate voltage can be applied to each layer. In the case of a 2-phase system, even with applied voltage, no potential difference may occur, resulting in the absence of electrostatic force in certain cases. However, a 3-phase ES clutch consistently generates electrostatic force even when the voltage is switched. As a 4-phase ES clutch shares a structure similar to the 3-phase system, it does not offer additional force benefits. Therefore, employing a 3-phase actuator is considered the most rational choice.

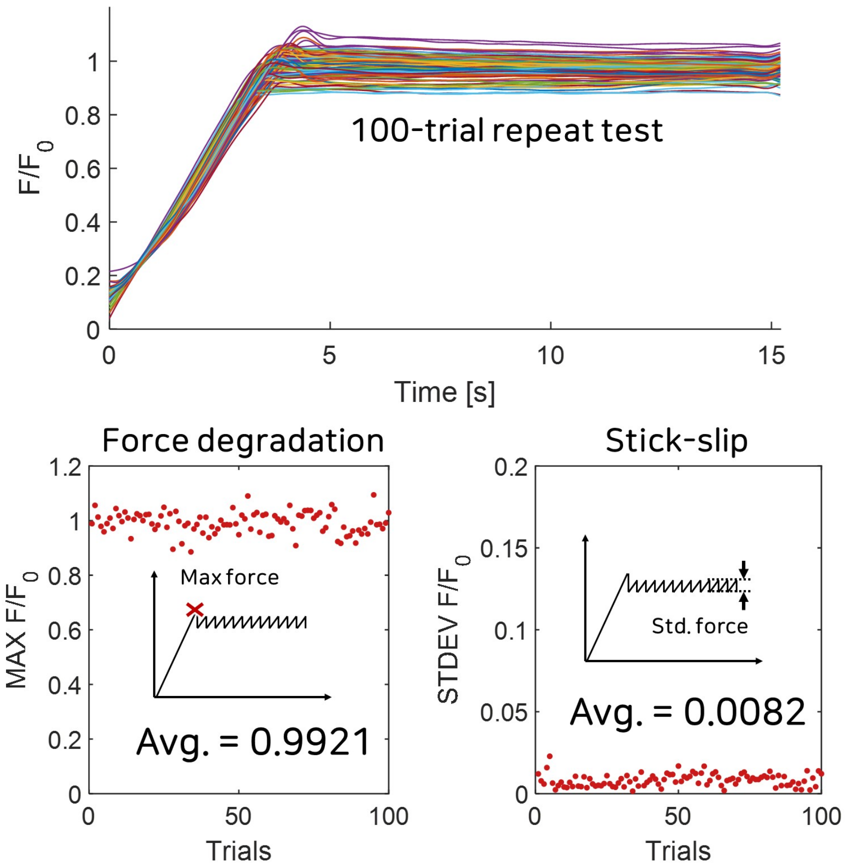

Figure S6: **100-trial repeat test.**

Repeated experiments were conducted on the same sample to evaluate the stability of the 3-phase ES clutch. Force degradation remained consistently close to unity, with an average value of 0.9921 measured over 100 cycles. The stick-slip behavior was also suppressed, maintaining a low and stable average value of 0.0082. These results confirm that the application of 3-phase voltage enables stable and consistent force generation throughout repeated operation.

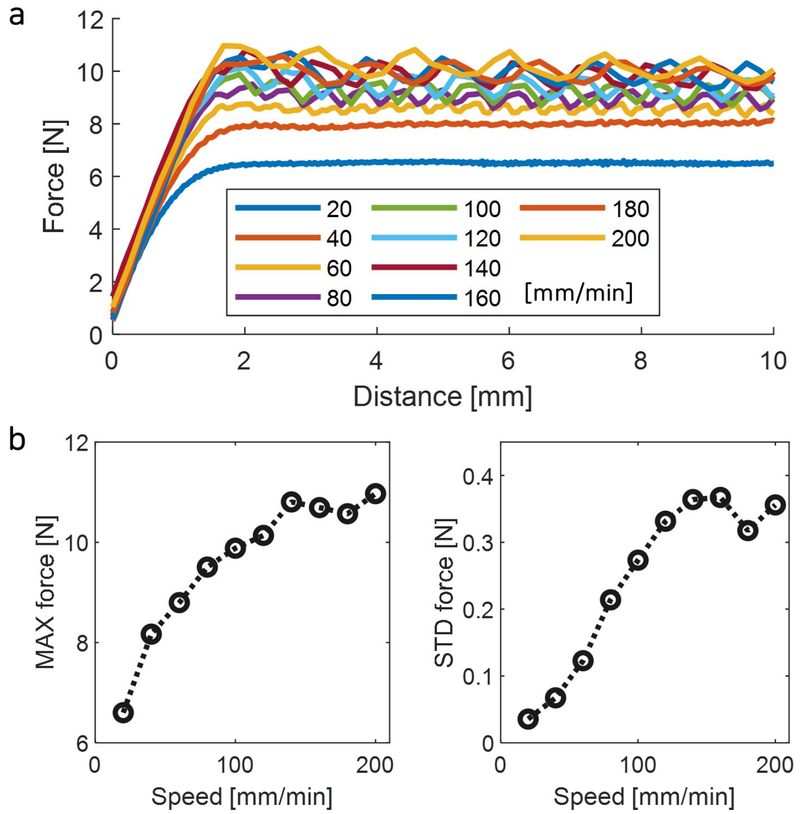

Figure S7: **3-phase ES force affected by external force speed.**

The response of the actuator to varying AC voltage frequencies presents a challenge, primarily due to the pulling speed of the CNC machine. The rate at which the CNC pulls to its maximum force is outpaced by the frequency changes of the actuator, making it difficult to analyze the maximum force of the actuator. Consequently, the CNC must be pulled faster to reach its maximum force in a shorter time frame. However, the rapid speed of the CNC leads to fluctuating maximum forces, resulting in inconsistent force measurements and an increased error.

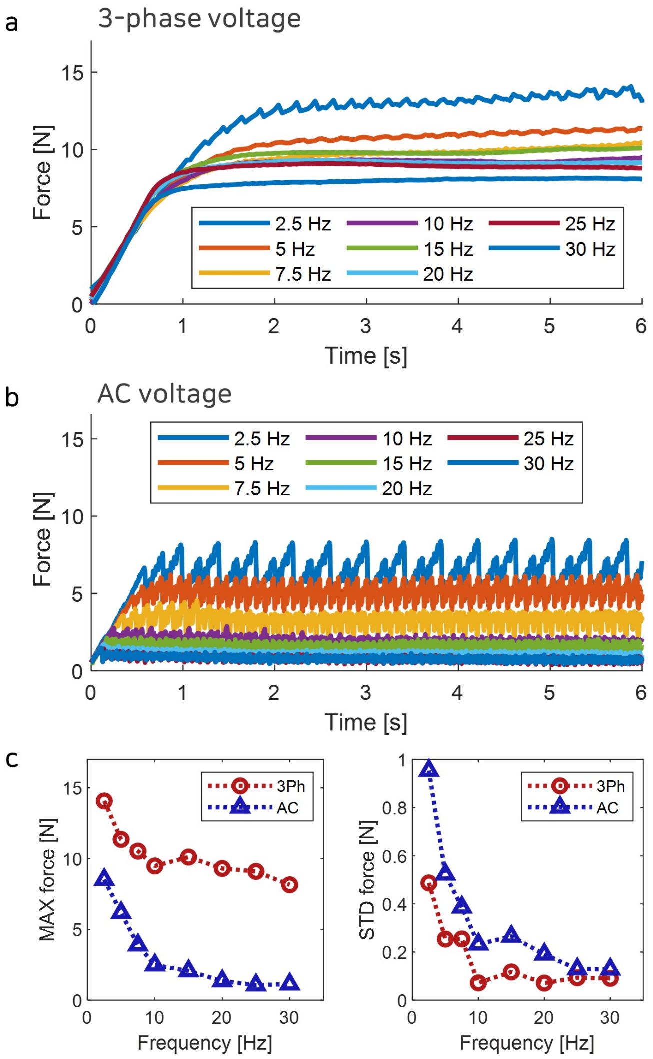

Figure S8: **ES force affected by 3-phase and AC voltage frequency.**

As the frequency increases, there is a noticeable reduction in the maximum force exerted by the actuator. At 1 Hz, variations in the magnitude of the force can be observed depending on the direction of the applied voltage. At 30 Hz, the actuator behaves like a filter, resulting in a decrease in the applied voltage. Furthermore, the electrostatic force dissipates before reaching its maximum, leading to an even further reduction in force. When a frequency of 30 Hz is applied, the high frequency creates a low-pass filter effect due to the actuator. This results in a lower voltage output and insufficient time for the actuator to fully charge and discharge. Therefore, high-frequency signals are not suitable for actuation, as they do not provide adequate charging and discharging periods for the actuator.

Supplementary Table

**Voltage type**	**DC**	**AC**	**3-Phase**
Total area (A_{total})	90 cm^2	90 cm^2	90 cm^2
Active area (A)	90 cm^2	90 cm^2	60 cm^2
Thickness (d)	10 μm	10 μm	10 μm
Dielectric constant; ε_r ($\varepsilon_0 = 8.542 \times 10^{-12}$)	3.4	3.4	3.4
Coefficient of friction; μ	0.6 (Static)	0.15 (Kinetic)	0.15 (Kinetic)
Frequency (f)	-	1 Hz	10 Hz
Theoretical Force (F)	129.6 N	32.4 N	21.6 N
Measured Force (F)	130. N	32.5 N	21.7 N
Theoretical force density (F/A_{total})	1.44 N/cm^2	0.36 N/cm^2	0.24 N/cm^2
Measured force density (F/A_{total})	1.63 N/cm^2	0.33 N/cm^2	0.25 N/cm^2
Theoretical power consumption (P)	-	4.18 mW	41.8 mW
Measured power consumption (P)	-	8.05 mW	76.2 mW
MAX F/F_0 at 5th trial (Force degradation)	0.65	0.88	0.99
STDEV F/F_0 at 5th trial (Stick-slip)	0.07	0.07	0.007

Table S1: **Comparison of DC, AC, and 3-Phase ES clutch**
